# Supplementary material for: Postoperative Circulating Tumor DNA Can Predict High Risk Patients with Colorectal Cancer Based on Next-Generation Sequencing
Source: Cancers (Basel). 2021 Aug 20;13(16):4190. doi: 10.3390/cancers13164190 (PMC8391973; doi:10.3390/cancers13164190)
Supplement: Supplementary file 1 [file cancers-13-04190-s001.zip › cancers-1321037-supplementary.pdf]

cancers

MDPI

Supplementary Files

Postoperative Circulating Tumor DNA Can Predict High Risk Patients with Colorectal Cancer Based on Next-Generation Sequencing

Chul-Seung Lee, Hoon-Seok Kim, Jeoffrey Schageman, In-Kyu Lee, Myungshin Kim and Yonggoo Kim

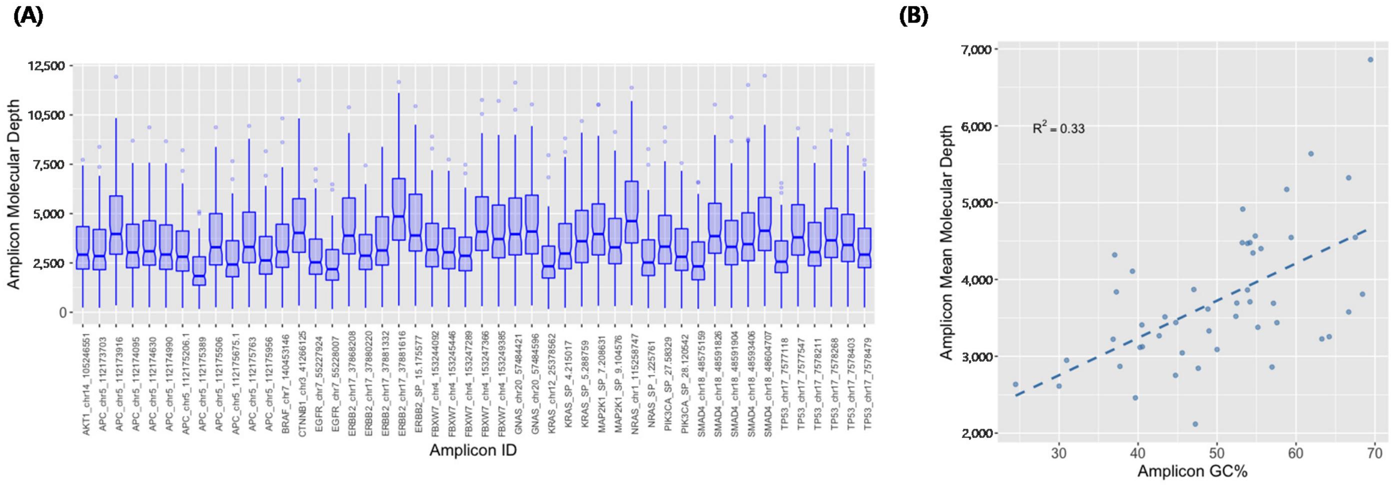

Figure S1. Quality control metrics of cell-free DNA assay. (**A**) Molecular coverage by each amplicon. (**B**) Scatter plot showing distribution of molecular depth by GC contents.

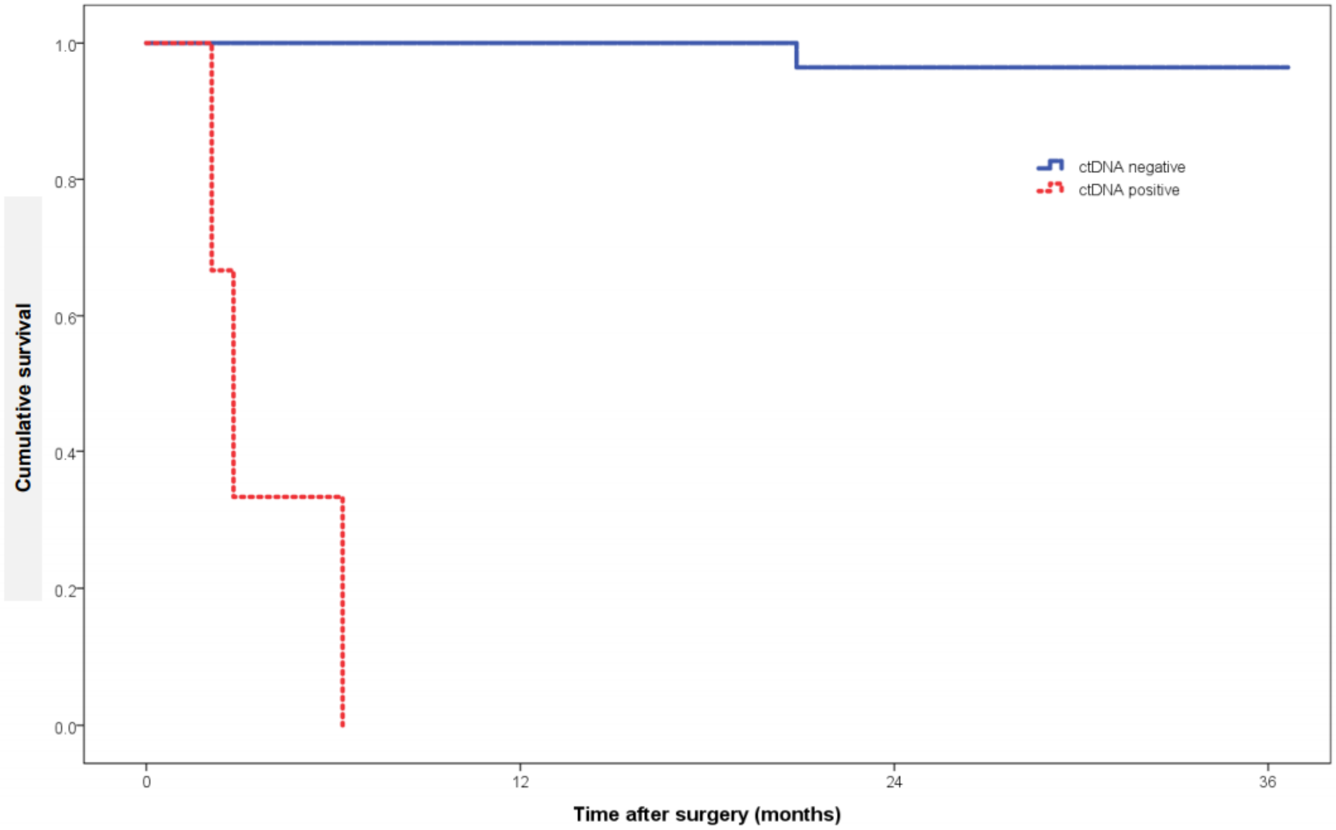

Figure S2. Disease free survival (DFS) stratified by postoperative circulating tumor DNA (ctDNA) status. Kaplan-Meier curve of DFS in 32 patients with R0 resection who had postoperative plasma samples.

Table S1. Detected variants of ctDNA in plasma samples of colorectal cancer patients

Patient No.	Chr	Position (Hg19)	Gene	Nucleotide Change	Amino Acid Change	Pre-op VAF(%)	Post-op VAF(%)	Post-chemo VAF(%)
11	chr17	7577538	*TP53*	c.743G > A	p.R248Q	0.27	0.23	0.00
11	chr4	153249385	*FBXW7*	c.1393C > T	p.R465C	0.50	0.32	0.00
40	chr17	7577539	*TP53*	c.742C > T	p.R248W	0.50	3.41	NA
40	chr5	112175751	*APC*	c.4463T > A	p.L1488*	0.94	2.26	NA
40	chr12	25398284	*KRAS*	c.35G > A	p.G12D	1.23	5.07	NA
15	chr17	7577539	*TP53*	c.742C > T	p.R248W	0.31	0.57	NA
15	chr5	112173917	*APC*	c.2626C > T	p.R876*	0.39	0.31	NA
2	chr17	7577121	*TP53*	c.817C > T	p.R273C	16.82	0.00	0.00
2	chr5	112175162	*APC*	c.3871C > T	p.Q1291*	7.22	0.00	0.00
2	chr4	153249385	*FBXW7*	c.1393C > T	p.R465C	5.53	0.00	0.00
33	chr17	7577094	*TP53*	c.844C > T	p.R282W	6.44	0.00	0.00
33	chr5	112175207	*APC*	c.3919del	p.I1307*	15.75	0.00	0.00
25	chr17	7577153	*TP53*	c.785G > T	p.G262V	8.15	0.00	0.00
28	chr17	7578407	*TP53*	c.523C > T	p.R175C	0.86	0.00	0.00
28	chr17	7577538	*TP53*	c.743G > A	p.R248Q	0.27	0.00	0.00
28	chr12	25398281	*KRAS*	c.38G > A	p.G13D	1.74	0.00	0.00
28	chr4	153247289	*FBXW7*	c.1513C > T	p.R505C	1.96	0.00	0.00
28	chr3	178936094	*PIK3CA*	c.1636C > A	p.Q546K	1.49	0.00	0.00
48	chr17	7578406	*TP53*	c.524G > A	p.R175H	3.16	0.00	0.00
17	chr17	7577121	*TP53*	c.817C > T	p.R273C	0.69	0.00	0.00
44	chr17	7577094	*TP53*	c.844C > T	p.R282W	0.17	0.00	0.00
44	chr12	25398284	*KRAS*	c.35G > C	p.G12A	0.13	0.00	0.00
46	chr17	7577538	*TP53*	c.743G > A	p.R248Q	0.06	0.00	0.00
46	chr4	153247289	*FBXW7*	c.1513C > T	p.R505C	0.08	0.00	0.00
35	chr17	7577539	*TP53*	c.742C > T	p.R248W	0.10	0.00	0.00
22	chr5	112173704	*APC*	c.2413C > T	p.R805*	0.55	0.00	0.00
42	chr5	112175207	*APC*	c.3919del	p.I1307*	0.12	0.00	0.00
20	chr1	115256530	*NRAS*	c.181C > A	p.Q61K	1.00	0.00	0.00
4	chr17	7577548	*TP53*	c.733G > A	p.G245S	0.19	0.00	NA
4	chr12	25398284	*KRAS*	c.35G > T	p.G12V	0.21	0.00	NA
31	chr17	7577094	*TP53*	c.844C > T	p.R282W	0.14	0.00	NA
31	chr12	25398281	*KRAS*	c.38G > A	p.G13D	0.20	0.00	NA
21	chr5	112175507	*APC*	c.4219_4220del	p.S1407*	0.45	NA	0.00
30	chr17	7578406	*TP53*	c.524G > A	p.R175H	0.10	NA	NA
50	chr17	7577539	*TP53*	c.742C > T	p.R248W	6.62	8.96	0.00
50	chr5	112175639	*APC*	c.4348C > T	p.R1450*	7.49	9.18	0.00
50	chr12	25398284	*KRAS*	c.35G > A	p.G12D	7.08	9.32	0.00
50	chr18	48591918	*SMAD4*	c.1081C > T	p.R361C	2.10	0.11	0.00
8	chr17	7578190	*TP53*	c.659A > G	p.Y220C	27.48	6.11	NA
26	chr5	112175171	*APC*	c.3880C > T	p.Q1294*	6.30	NA	NA

NA, not available; VAF, variant allele frequency; ctDNA, circulating tumor DNA; Pre-op, 2 days before surgery; Post-op, 10 days after surgery; Post-chemo, the end day of the last chemotherapy.
